# Effect of Hot Deformation Parameters on Heat-Treated Microstructures and Mechanical Properties of 300M Steel

**DOI:** 10.3390/ma15248927

**Published:** 2022-12-14

**Authors:** Fei Du, Peng Zhou, Peng Guo, Cheng Li, Lei Deng, Xinyun Wang, Junsong Jin

**Affiliations:** 1 State Key Laboratory of Materials Processing and Die & Mould Technology, Huazhong University of Science and Technology, 1037 Luoyu Road, Wuhan 430074, China; 2 Huazhong University of Science & Technology Analytical & Testing Center, 1037 Luoyu Road, Wuhan 430074, China; 3 State Key Laboratory of Advanced Brazing Filler Metals and Technology, Zhengzhou Research Institute of Mechanical Engineering Co., Ltd., Zhengzhou 450001, China

**Keywords:** 300M steel, hot deformation, heat treatment, microstructure evolution, yield strength

## Abstract

The high strength of 300M steel originates from the heat treatment process after forging, but how hot deformation affects the heat-treated microstructure and mechanical properties is unclear. In this study, compression tests under different hot deformation parameters and post-deformation heat treatment experiments were carried out, and the martensite transformation process was investigated using in situ observation. The results show that the grain size of the specimen deformed at low temperature and high strain rate is smaller, and annealing twins will be formed. Both austenite grain boundaries and twin boundaries hinder the growth of martensite blocks, reducing the size of martensite units after heat treatment and thus resulting in higher yield strength. Besides, the mathematical models were established to describe the relationship between hot deformation parameters and grain size after deformation, martensite packet size and martensite block width, respectively, after heat treatment. The relationship between yield strength and hot deformation parameters was also analyzed. According to the results and models, the hot deformation parameters would be optimized more reasonably to improve the final mechanical properties of 300M steel forgings.

## 1. Introduction

As one of the ultra-high strength steels, 300M steel has good fracture toughness, excellent stress corrosion resistance and fatigue resistance, and is widely used for key parts in the aviation industry and other fields, such as aircraft landing gear, pressure vessels, fasteners, etc. [1,2,3]. Generally, the manufacturing of such large bearing forgings consists of conducting hot deformation first, then heat treating and, finally, machining [4]. The hot deformation process is not only a process to change the shape and size of forgings, but also can control and improve the final microstructure of 300M steel in combination with the heat treatment process [5,6]. Therefore, it is crucial to study the changes in the structure and properties of 300M steel throughout the process, including hot deformation and heat treatment, to ensure the excellent service performance of 300M steel.

Several scholars have studied the hot deformation and heat treatment process of 300M steel. For the hot deformation process, scholars mainly focus on the rheological and recrystallization behavior under different deformation parameters. Skubisz et al. [7] investigated the influence of processing conditions on the forgeability, microstructure and properties of 300M steel, and dynamic behavior modeling and processing maps were carried out. Qi et al. [8] studied the deformation behavior of 300M steel at 850~1200 °C and 0.001~10 s^−1^ and found that temperature and strain rate significantly affect the microstructure evolution. Luo et al. [9] studied the microstructure evolution behavior of 300M steel under different deformation parameters by isothermal compression experiments, and the results showed that fine equiaxed recrystallized grains formed near the initial grain boundaries at a certain amount of deformation when the strain rate was 25 s^−1^. Liu et al. [10] investigated the effect of hot deformation parameters on the dynamic recrystallization behavior of 300M steel and developed a kinetic model of dynamic recrystallization based on the flow stress–strain curve. Guo et al. [11] investigated the dynamic recrystallization behavior of 300M steel at deformation temperatures of 900–1150 °C and strain rates of 0.001–50 s^−1^ and found that the dynamic recrystallization mechanism of 300M steel was closely related to the strain rate.

Since the phase transformation during heat treatment determines the final mechanical properties of 300M steel, the research on heat treatment has focused on phase transformation and its effect on mechanical properties. Liu et al. [12] investigated the characteristics of martensite transformation from deformed austenite with various states of 300M steel. The results showed that deformed-quenched 300M steel at different strains mainly consisted of lath martensite, complemental twinned martensite and retained austenite, and the length and width of the martensite block decreased with the increase of strain. In addition, Kasana et al. [13] investigated the effects of four different heat treatment routes on the properties of 300M steel and successfully developed 300M steel with minimum segregation and superior mechanical properties. Owing to non-uniform phase formation of 300M large forgings in the quench process, Chentouf et al. [14] studied the influence of different cooling rates on phase transformation and determined the critical transition temperature and microstructure accurately. However, these studies did not explain the influence mechanism of deformation parameters on the martensite transformation, nor did they consider the effect of strain rate. In addition, some scholars [15,16,17] investigated the effect of prior austenite grain size on martensite unit size and established a relationship model between them. However, these studies were conducted to obtain different austenite grain sizes by heat treatment, which did not take into account the influence of dislocation, twin and other factors introduced in hot deformation. In summary, the current studies on hot deformation and heat treatment of 300M steel are independent. In actual production, the service performance of large bearing forgings is determined by the combination of hot deformation and subsequent heat treatment. In this work, the whole process of hot deformation and heat treatment was studied for the first time to help understand the inheritance relationship between them and obtain the optimal process parameters for excellent forgings.

In this study, through the hot compression and heat treatment experiments of 300M steel, the effect of hot deformation parameters on heat-treated microstructure and mechanical properties was studied. The martensite transformation during the cooling process of heat treatment was analyzed by in situ observation, and the influence mechanism of hot deformation parameters on the heat-treated microstructure was clarified. In addition, the relationship models between hot deformation parameters and grain size after deformation, martensite packet size and martensite block width after heat treatment were established. The relationship between yield strength and hot deformation parameters was also analyzed.

## 2. Materials and Methods

**Figure 1:**
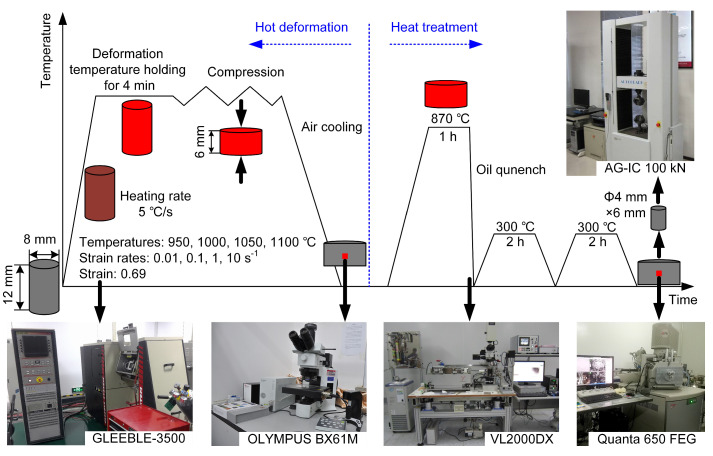
Experimental procedure.

The material used in this study was commercial 300M steel, and its chemical composition (wt.%) is shown in Table 1. The initial material is a bar with a diameter of 300 mm and a length of 1500 mm. The experimental procedure is shown in Figure 1. Firstly, isothermal compression experiments were conducted on the specimen of φ 8 mm×12 mm using the GLEEBLE-3500 thermal simulation tester. The deformation temperatures were 950 °C 1000 °C 1050 °C and 1100 °C, the strain rates were 0.01 s^−1^, 0.1 s^−1^, 1 s^−1^ and 10 s^−1^ and the compression amount was 50% (corresponding to the true strain of 0.69). During the compression experiments, the specimens were heated to the deformation temperature at a heating rate of 5 °C·s^−1^, held for 4 min, then compressed isothermally and air-cooled to room temperature. The compressed specimens were subsequently heat treated. Firstly, the specimens were austenitized at 870 °C for 1 h, then oil quenched and twice tempered at 300 °C for 2 h.

After hot deformation and heat treatment, the specimens were cut along the axial direction and polished. The hot deformation specimens were etched by saturated picric acid solution at room temperature, and the grain structure was observed by a metallographic microscope (OLYMPUS BX61M). The heat-treated specimens were etched with a 4% nitric acid alcohol solution, and the microstructure was observed by scanning electron microscope (SEM). The distribution of martensite units and residual austenite were obtained by electron backscattered diffraction (EBSD) with a scanning step of 0.1 μm. In order to visualize the microstructure evolution during the quenching process of heat treatment, an in situ laser confocal microscope (VL2000DX) was used to observe the martensite transformation during cooling, with an image acquisition frequency of 5 images/s.

A sample of φ 4 mm×6 mm was taken from the heat-treated specimens by wire cutting, and a compression test was performed at room temperature using an AC-IC 100 kN mechanical tester to obtain the yield strength.

## 3. Results and Discussion

### 3.1. Microstructure

**Figure 2:**
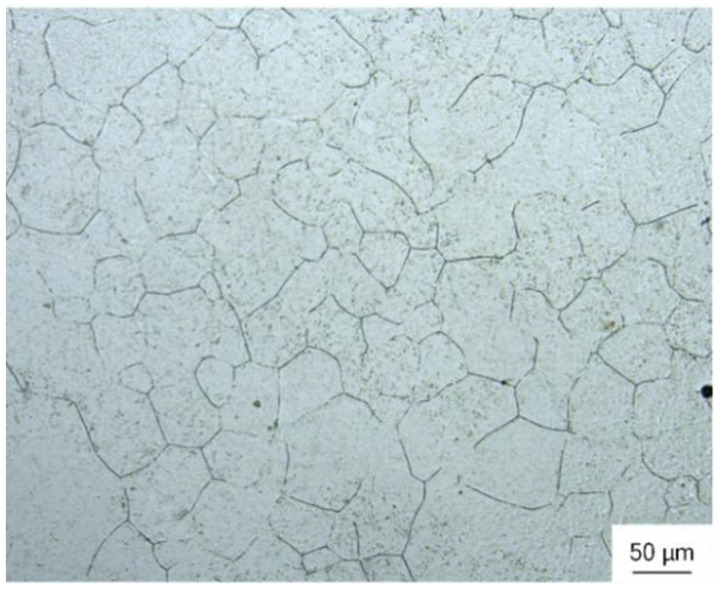
Initial grain structure.

**Figure 3:**
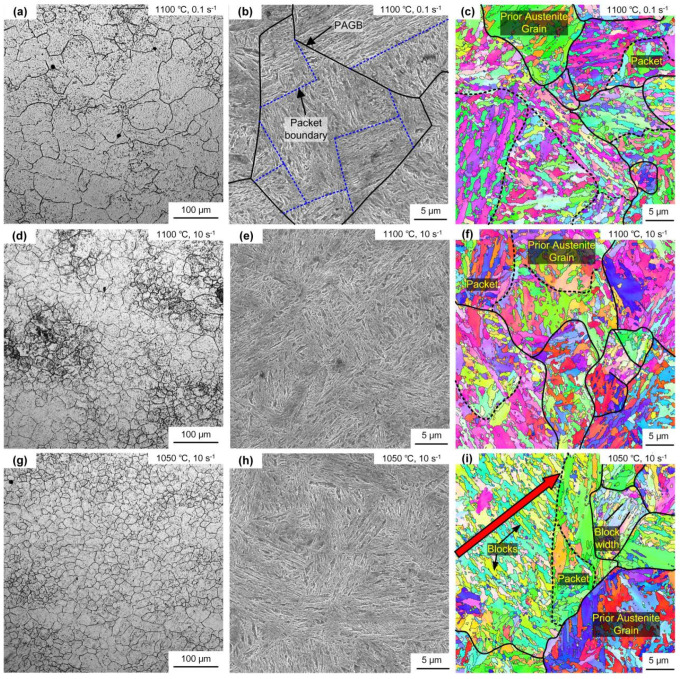
Microstructure under different hot deformation parameters: (**a**,**d**,**g**) are the grain structures after deformation; (**b**,**e**,**h**) are SEM of martensite microstructure after heat treatment (the dotted lines represent martensite packet boundaries); (**c**,**f**,**i**) are EBSD of martensite microstructure after heat treatment (the dotted lines represent martensite packet boundaries).

**Figure 4:**
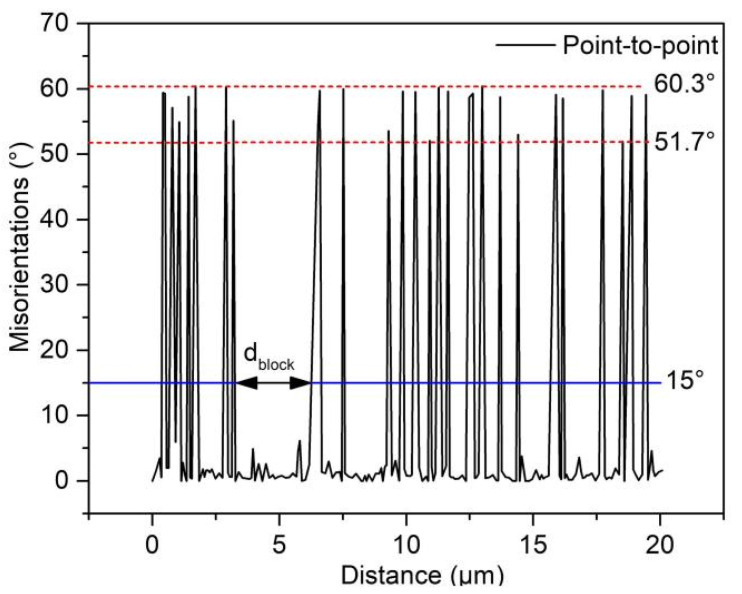
Point-to-point orientation difference on the red-colored line in Figure 3i.

The initial state of 300M steel is annealed, and its metallographic structure is shown in Figure 2, with an initial grain size of about 50 μm, and the microstructure of 300M steel under different deformation parameters is shown in Figure 3. The microstructure is affected by temperature and strain rate [18,19]. Figure 3a,d,g show the metallographic images after hot deformation. It can be seen that dynamic recrystallization occurred under different hot deformation conditions, and the recrystallized grain size decreased with the increase of the Zener–Hollomon (*Z*) parameter. The *Z* parameter is the strain rate coefficient coupled with the temperature effect, with the expression $Z\;=\;\dot{\varepsilon }\;\exp\left(Q/RT\right)$ [18], where *Q* is the deformation activation energy (381.34 kJ·mol^−1^) [11], *R* is the gas constant (8.314 J·(mol·K)^−1^) and *T* is the thermodynamic temperature. Figure 3b,e,h show the SEM of the heat-treated specimens, and the prior austenite grain boundaries (PAGB), martensite packets and blocks were observed obviously. Figure 3c,f,i show the EBSD of the heat-treated specimens, and the black lines in the figures are the interfaces with orientation differences of 21–47°, which can be considered as PAGB during heat treatment [20]. Figure 4 shows the point-to-point orientation difference on the red line in Figure 3i. The interface with a point-to-point orientation difference greater than 15° is usually considered a martensite grain boundary. As shown in Figure 4, the orientation difference of the martensite block grain boundaries is between 51.7° and 60.3°. The width of the martensite block can be obtained by measuring the distance between adjacent grains at 15° (blue line in Figure 4).

**Figure 5:**
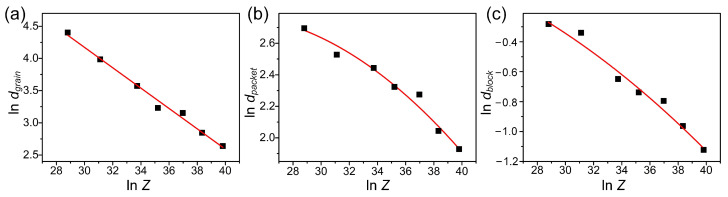
Microstructure size versus *Z* parameter: (**a**) grain size versus *Z* parameter after hot deformation; (**b**) martensite packet size versus *Z* parameter after heat treatment; (**c**) martensite block width versus *Z* parameter after heat treatment.

Based on the microstructure and EBSD results, the linear intercept method [21] was used to calculate the recrystallized grain size (*d_grain_*), martensite packet size (*d_packet_*) and martensite block width (*d_block_*) under different hot deformation parameters, as shown in Figure 5. It can be seen that ln *d_grain_*, ln *d_packet_* and ln *d_block_* decreased with the increase of ln *Z*. Among them, the relationship between ln *d_grain_* and ln *Z* is linear, and the relationship model between recrystallized grain size after hot deformation and *Z* parameters was established by linear fitting, as shown in Equation (1). In Figure 5b,c, when ln *Z* > 36, the decrease tendency of ln *d_packet_* and ln *d_block_* increased with the increase of ln *Z*. The relationship models between the martensite unit size after heat treatment and *Z* parameters were established respectively by data fitting, as shown in Equations (2) and (3). The reasons for this phenomenon will be analyzed in Section 3.2.

*d_grain_* = 3.54 × 10^4^ × *Z*^−0.2138^ (1)

*d_packet_* = 3.4 × *Z*^0.137−0.003ln *Z*^ (2)

*d_block_* = 1.1 × *Z*^0.035−0.0016ln *Z*^ (3)

### 3.2. Mechanism of Microstructure Evolution

**Figure 6:**
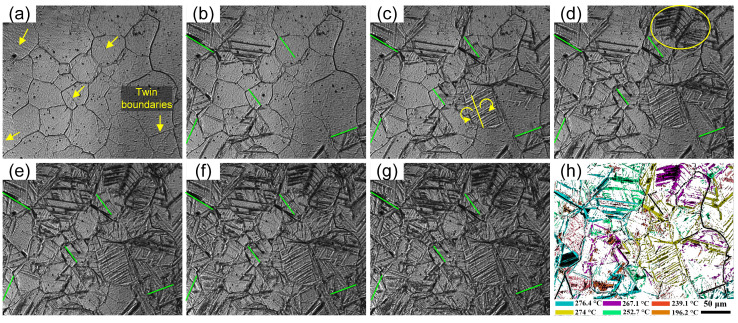
In situ observation of the martensite transformation of 300M steel during the cooling process at (**a**) 279.3 °C (the yellow arrows represent twin boundaries); (**b**) 276.4 °C (the twin boundaries in Figure 6b,c are represented by green lines); (**c**) 274 °C (the yellow arrows represent phase transition moment); (**d**) 267.1 °C; (**e**) 252.7 °C; (**f**) 239.1 °C; (**g**) 196.2 °C; and (**h**) the superposition of martensite formed at different temperatures (the different colors represent martensite transformed at different temperatures, and the black lines represent the austenite grain boundaries and twin boundaries).

**Figure 7:**
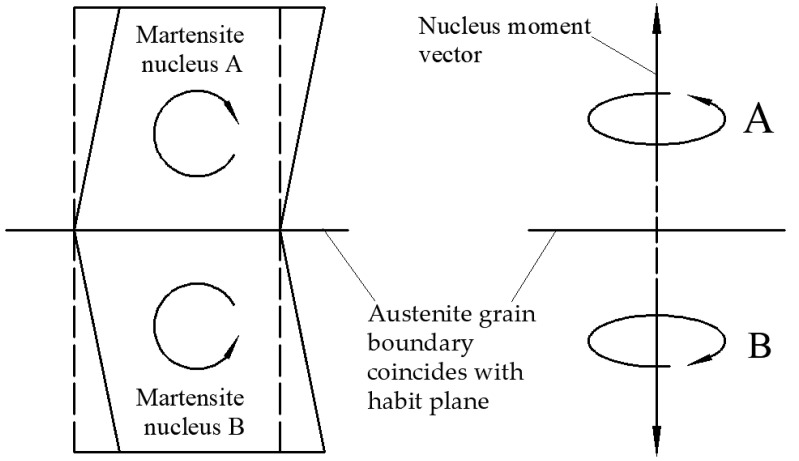
Schematic diagram of the moments during martensite nucleation.

The martensite transformation during the cooling process of heat treatment is shown in Figure 6. The specimen to be observed was pre-compressed at 1050 °C, 10 s^−1^ (ln *Z* = 37). Austenite grains were nearly equiaxed. In addition, austenite grain boundaries and twin boundaries can be clearly seen in Figure 6a when the temperature was above the martensite transformation point. No twins were observed after the hot deformation, as shown in Figure 3g. The twins in Figure 6a were annealing twins generated during the holding process. When the temperature was lowered to the martensite transformation point, martensite blocks started to form inside the austenite grains. First, some martensite blocks with different orientations divided an austenite grain into 3–5 parts. The supercooling degree increased with the further decrease in temperature, and the undercooled austenite continuously transformed to martensite. The martensite blocks with the same habit plane constituted a martensite packet, as shown in Figure 6b,c. From the results of in situ observation, the martensite blocks and packets did not grow through austenite grain boundaries. Some ended up growing at other martensite interfaces in the same parent austenite grain, and some ended up growing at austenite grain boundaries. It is obvious from Figure 6h that the colored areas representing the martensite blocks do not cross the black lines representing the austenite grain boundaries. Exceptionally, in Figure 6c,d, it appears that the martensite block “crosses” the austenite grain boundary, but this austenite grain boundary is probably the habit plane for martensitic transformation. Martensite and austenite are different in atomic arrangement and crystal structure; when the martensite nucleus A is perpendicular to the habit plane generated in austenite, the martensite nucleus A will exert a phase transition moment on the austenite crystal. In order to reduce the nucleation work and eliminate the rotational effect caused by the phase transition moment on the austenite, the material will form the martensite nucleus B perpendicular to the habit plane on the other side of the habit plane so that the moment vectors of nucleus B and A offset each other, as shown in Figure 7. When the habit plane is just at the grain boundary, there is a phenomenon that the martensite “crosses” the grain boundary. In other words, two different martensite blocks are on both sides of the habit plane.

In addition, as shown in Figure 6b,c, the directions of martensite blocks were different on both sides of twin boundaries. That is to say, the martensitic blocks also did not cross the twin boundaries, except the austenite grain boundaries, indicating that the twin boundaries located inside the austenite grains also hinder the growth of martensite. When the *Z* parameter increased, the grain sizes decreased during the hot deformation process. It is generally believed that distortion energy can promote the formation of annealing twins [22]. As the *Z* parameter increased further (ln *Z* > 36), the distortion energy of the sample increased greatly, which led to the formation of annealing twins during the holding process. The twin boundaries and austenite grain boundaries simultaneously hindered the growth of martensite blocks during heat treatment, resulting in an increasing tendency to reduce the martensite packet size and block width.

**Figure 8:**
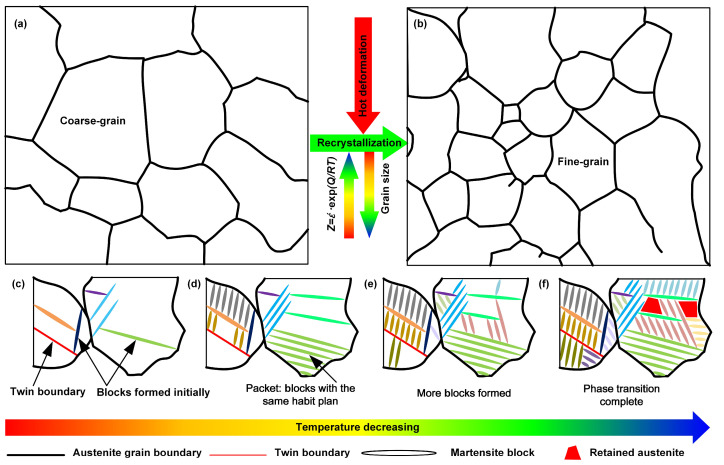
Schematic diagram of the martensite transformation mechanism: (**a**) initial grains; (**b**) formation of fine grains after deformation; (**c**) division of austenite grains by martensite blocks and annealing twins; (**d**) formation of martensite packets by martensite blocks with the same habit plane; (**e**) increase in martensite content; (**f**) completion of phase transformation.

Based on the above analysis, the mechanism of hot deformation parameters on the martensite unit size after heat treatment is proposed in Figure 8. First, the initial structure undergoes dynamic recrystallization during hot deformation, and the initial coarse grains are refined. With the increase of the *Z* parameter, the recrystallized grain size decreases, as shown in Figure 8a,b, and annealing twins are formed during the holding process. During the cooling process, several martensite blocks are formed first in the austenite grains, which divide the austenite grains into 3–5 parts because the growth of martensite blocks will not cross the austenite grain boundaries and twin boundaries, as shown in Figure 8c. As the temperature decreases further, the austenite continues to transform to martensite along the previously formed martensite block habit plane, and these martensite blocks with the same habit plane will form a martensite packet, as shown in Figure 8d,e. When the martensite transformation is completed, 3–5 martensite packets are generated in one austenite grain. In addition, the untransformed austenite exists between the martensites as residual austenite, as shown in Figure 8f.

### 3.3. Mechanical Properties

**Figure 9:**
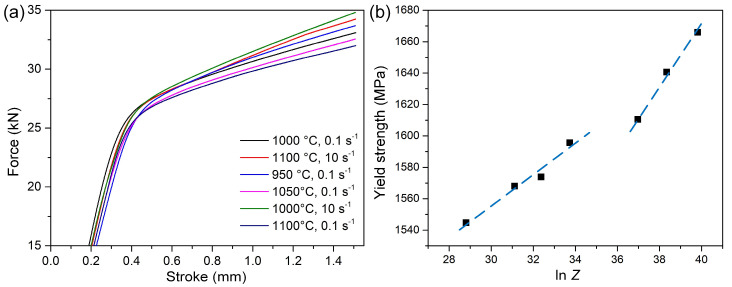
The results of the compression tests at room temperature: (**a**) Force–stroke curve during compression; (**b**) yield strength versus *Z* parameters.

The compression curves of the heat-treated specimens at room temperature are shown in Figure 9a. It can be seen that the hot deformation parameters have a significant effect on the mechanical properties after heat treatment. The force increased rapidly with the increase of stroke. When the applied stress reached the yield strength, the material entered plastic deformation. The yield strength of heat-treated specimens gradually increased with the decrease of deformation temperature or the increase of strain rate. Figure 9b shows the relationship between yield strength (*σ_y_*) and *Z* parameters. Based on previous studies, *σ_y_* can be described by the following equation [16,23]:

*σ_y_* = *σ*_0_ + *σ_p_* + *σ_s_* + *σ_ρ_* + *k_HP_d*^−1/2^ (4)

where *σ*_0_ is the friction stress for pure iron, *σ_p_* is the precipitation hardening, *σ_s_* is the solid solution hardening, *σ_ρ_* is the hardening of dislocations and *k_HP_d*^−1/2^ is the grain boundary strengthening (*k_HP_*: Hall–Petch slope; *d*: the effective grain size or the spacing of high angle boundaries). In the heat treatment process, the deformed sample was held for a long time above the recrystallization temperature, making the dislocation density greatly decrease and remain stable. In addition, the cooling rate was sufficient to make almost all austenite transform into martensite [24]. Meanwhile, due to the same concentration of alloy elements and heat treatment process, the first four terms on the right side of Equation (4) are expected to be nearly constant [16,25]. Therefore, the increase of yield strength is mainly caused by grain boundary strengthening. Since the martensite block is a high angle boundary, the yield strength is directly related to *d_block_*. As discussed in Section 3.2, during the hot deformation process, the grain size decreased with the increase of the *Z* parameter. Therefore, the yield strength increased with the increase of the *Z* parameter. In addition, when ln *Z* > 36, annealing twins were formed during the heat treatment. The twin and austenite grain boundaries simultaneously hindered the growth of the martensite block, which had a stronger inhibition on the growth of martensite. Accordingly, the increasing tendency of yield strength increased, as shown by the blue dotted line in Figure 9b.

## 4. Conclusions

In this study, the effect of hot deformation parameters on heat-treated microstructure and mechanical properties was investigated through hot compression and heat treatment tests of 300M steel. In addition, in situ observation of the martensite transformation during the cooling process of heat treatment was carried out. The following conclusions were obtained.

(1) Dynamic recrystallization occurred after hot deformation, and the microstructure after heat treatment was mainly martensite. With the increase of the *Z* parameter, the recrystallization grain size and the martensite unit size decreased. In addition, the decreasing tendency of the martensite block width and the martensite packet size also increased.
(2) With the increase of the *Z* parameter, annealing twins were formed during the heat treatment. The twin boundaries hinder the growth of martensite, making the decreasing tendency of the martensite unit size increase.
(3) The yield strength was mainly affected by the martensite unit size and increased with the increase of the *Z* parameter. When ln *Z* > 36, annealing twins were formed and the increasing tendency of yield strength increased.

## Figures and Tables

**Table 1:** The chemical composition of 300M steel (wt.%).

C	Mn	Si	Ni	Cr	V	Mo	Fe
0.38	0.74	1.64	1.87	0.84	0.08	0.40	Balance

## Data Availability

The data presented in this study are available on reasonable request from the corresponding author.
